# Aqueous extract of dioscorea opposita thunb. normalizes the hypertension in 2K1C hypertensive rats

**DOI:** 10.1186/1472-6882-14-36

**Published:** 2014-01-21

**Authors:** Nurmuhammat Amat, Raziya Amat, Sajida Abdureyim, Parida Hoxur, Zulpiya Osman, Dolkun Mamut, Anake Kijjoa

**Affiliations:** 1 Traditional Uighur Medicine Institute, Xinjiang Medical University, 830011 Urumqi, Xinjiang, PR China; 2 First Affiliated Hospital, Xinjiang Medical University, 830011 Urumqi, Xinjiang, PR China; 3 Traditional Chinese Medicine Hospital, Xinjiang Medical University, 830011 Urumqi, Xinjiang, PR China; 4 Salamat Biotechnology Co., Ltd, Urumqi, Xinjiang 830011 PR China; 5 ICBAS-Instituto de Ciências Biomédicas de Abel Salazar and CIIMAR, Universidade do Porto, Rua de Jorge Viterbo Ferreira, 228, 4050-313 Porto, Portugal

**Keywords:** Antihypertensive, *Dioscorea opposite* Thunb, 2K1C experimental hypertension, Angiotensin-II, Endothelin-1, Hypertrophy

## Abstract

**Background:**

Dioscorea opposita Thunb. (Huai Shan Yao, DOT), a common staple food in China, has been used for more than 2000 years in traditional Chinese medicine (TCM) to treat different systemic diseases including hypertension. The objective of this study was to investigate the possible antihypertensive effects of the aqueous extract of (DOT) in renovascular hypertensive rats as well as the mechanism in reducing blood pressure.

**Methods:**

The two-kidney one-clip (2K1C) Goldblatt model of renovascular hypertension was used in Wistar rats. Rats with captopril, low-dose DOT and high-dose DOT treated 2K1C groups for 6 weeks. The blood pressure, cardiac mass index (heart weight/body weight), plasma level of angiotensin-II (Ang-II), endothelin-1(ET-1), superoxide dismutase (SOD) and malondialdehyde (MDA) were evaluated.

**Results:**

DOT significantly reduced mean systolic and diastolic blood pressure after treatment. DOT also significantly increased plasma SOD activity but decreased plasma MDA concentration. Renal function was improved with captopril and DOT. DOT reduced plasma Ang-II activity and plasma ET concentration. They couldalso significantly reduce the left ventricular hypertrophy and cardiac mass index.

**Conclusions:**

Our results suggest that DOT may have an antihypertensive effect on hypertension by inhibit ET-converting enzyme and antioxidant activity, which warrant further exploration.

## Background

Hypertension is the most common risk factor for myocardial infarction, stroke, heart failure, arterial fibrillation, aortic dissection and peripheral arterial diseases. It is among the most common chronic illnesses the world faces [1,2] and remains the leading cause of death worldwide and one of the world’s greatest public health problems. Although many new antihypertensive drugs with improved efficacy have been introduced to the market, they still possess serious side effects. On the one hand, nutrition and physical exercises are gaining more importance in the treatment of hypertension. On the other hand, attention has recently been focused on herbal and mineral preparations which are traditionally used as potential therapeutic agents in the prevention and management of cardiovascular diseases [3-6].

Chinese yam or *Shan yao* comprises various species of the genus *Diascorea,* which are widely cultivated in China and their tubers are used as food as well as for medicinal purposes. *Shan yao* has been considered as an important invigorant in traditional Chinese medicine (TCM) for many years [7]. However, the most important variety is *Dioscorea opposita* Thunb. or *Huai Shan Yao* in Chinese, which is used in TCM as a tonic for more than 2000 years. It is generally believed that an intake of the Chinese yam may be beneficial to improve the function of the spleen, stomach, kidney and lung. As a result, it is used clinically for the treatment of poor appetite, chronic diarrhea, asthma, dry cough, frequent or uncontrollable urination, diabetes and emotional instability [8,9]. (Chinese Pharmacopoeia, 2005 edition). The Chinese yam contains a variety of phytochemicals, including saponins, starch, mucopolysaccharides, protein, amino acids, mucilage, polyphenols *etc.*[10-13]. Modern research showed that yam extract has many physiological functions such as anti-diabetic, anti-hypercholesterolemia, anti-acetaminophen-induced hepatotoxicity and nephrotoxicity as well as antioxidant activity [12,14-17]. Interestingly, the water yam (*Dioscorea alata* L.), another species *Shan yao* has been shown to possess antihypertensive activity in hypertensive animal models [18], suggesting that consumption of fresh yam tubers has potential health benefits for human beings. Moreover, powdered and liquid yam products are nowadays extensively used in a variety of food products in China and countries in the Far East. Due to the increasing concern about the influence of foods on health condition, we have investigated the effect of the aqueous extract of untreated control group (DOT) on hypertension.

Of the various experimental or genetic models of hypertension, the Goldblatt chronic two-kidney, one-clip hypertension (2K1C) is a classical model of renovascular angiotensin-II-dependent hypertension. Experimental model of renal (Goldblatt) hypertension is one of the widely used models for the study of pathophysiology of hypertension and antihypertensive drugs [19]. The fact that the renin–angiotensin system (RAS) contributes critically to the pathophysiology of 2K1C Goldblatt hypertension is well established [20]. The 2K1C model, which exhibits a transient increase in the activity of RAS and a sustained rise in blood pressure, has been described as very close to human mature hypertension [21,22]. Thus, hypertension in this model is primarily the result of an augmented total peripheral resistance and, in mild cases of renal artery stenosis, bilateral reduction in renal-clearance function [23]. These physiological abnormalities are principally the result of a considerable increase in tissue and circulating levels, and direct actions of Ang-II [24]. Evidence shows that as the condition advances, the role of Ang-II in maintaining hypertension subsides, and other mediators become more effective in determining the level of blood pressure [25,26]. Therefore drugs acting on RAS are major factors in the treatment of hypertension. The occurrence of hypertension is related to many factors. A large number of clinical studies and animal experiments showed that there is a close relationship between hypertension and free radicals. In recent years, high blood pressure and oxidative stress became a focus for researchers. Compelling data from molecular and cellular experiments, together with animal studies, implicate a role of oxidative stress in hypertension [27] and thus oxidative stress induced by reactive oxygen species (ROS) could be involved in the pathogenesis of hypertension. ROS can influence vascular, renal, and cardiac function and structure by modulating cell growth, contraction/dilatation, and inflammatory responses via redox-dependent signaling pathways. Free radicals may participate in hypertension by damaging target organs through a variety of ways. Of the ROS and RNS families, superoxide anion (O_2_^-^), hydrogen peroxide (H_2_O_2_) and NO are of major importance in the cardiovascular and renal systems [28,29]. The generation of ROS is a cascade of reactions initiated by the formation of superoxide, which is rapidly dismutated to H_2_O_2_, either spontaneously or catalyzed by the enzyme superoxide dismutase (SOD) [30]. Therefore, the protection of endothelial from free radical injury is very important in treating hypertension.

So far, there is no report on the anti-hypertensive effects of DOT and the information regarding to the mechanism of DOT against hypertension is very scarce. For these reasons, we describe, in the present paper, the anti-hypertensive effects of the aqueous extract of DOT, together with changes in biological parameters such as plasma Ang-II, ET-1, SOD activity and MDA level, following the administration of DOT to the renal hypertensive rat model.

## Methods

### Material

The aqueous extract of *Dioscorea opposita* Thunb. (DOT) was provided by the Salome Biotechnology Co. Ltd, Urumqi, Xinjiang, PR China. Yield for the extract is 50 g dry extract per kg of dried *Dioscorea opposita* tuber. Dried DOT was dissolved in 0.9% saline to obtain a primary solution of 0.2 g/ml. The primary solution was diluted with 0.9% saline to obtain the various treatment solutions and administered under the same volume by daily gavage. Captopril obtained from Captopril, Sino Shanghai Shi Bao Co. Ltd.

### Animals

The animals used in the present study were adult male Sprague–Dawley rats (10–12 weeks old with body weight 180-200 g), obtained from the animal house of the Animal Center of Xinjiang Medical University (Urumqi, Xinjiang, PR China). The animals were housed in colony cages, under standard laboratory conditions (12 h light, 12 h dark cycle), with free access to standard commercial diet and water. All experimental procedures used in the present study were approved by the Ethics Committee of the Xinjiang Medical University which has adopted the guidelines established by the Xinjiang Uighur Autonomous Region on Animal Care and Experimentation.

### Induction of renovascular hypertension (two-kidney, one-clip model)

The Goldblatt 2K1C model of hypertension was induced according to the procedure described by Umar et al. [31]. Briefly, the rats were anesthetized with sodium pentobarbital (50 mg/kg, intraperitoneally). The left renal artery was exposed by retroperitoneal flank incision and dissected free of the renal vein and connective tissue. A silver clip with a lumen of 0.22 mm was placed around the artery for partial occlusion; in sham operations, the artery was not clipped. After 6 weeks the systolic blood pressure (SBP) was measured using the tail-cuff method in conscious rats. Only hypertensive rats (SBP above 150 mm Hg) were used in the experiments.

### Animal treatment

At 6 weeks after the surgery, when hypertension was established, the 2K1C rats were divided into five groups of 10–12 rats: groups 1 - sham-operated control rats (served as negative control); groups 2 - an untreated model group; groups 3 - rats treated with captopril (30 mg/kg BW per day); groups 4 - rats treated with low-dose DOT (DOT-L, 210 mg/kg BW per day); group 5- rats treated with high-dose DOT (DOT-H, 420 mg/kg BW per day), Rats were treated for 6 weeks with daily oral administration of the products or the same volume of vehicle (0.9% saline)(group 1 and group 2). Doses of DOT were chosen in reference to doses commonly used in human and doses used in previous experiments. All rats were weighed and their blood pressure was measured once a week for 4 weeks (Table 1).

**Table 1:** Cardiovascular parameters in two-kidney, one-clip Goldblatt hypertensive rats after 6 weeks of daily oral treatment with captopril (30 mg/kg per day), low-dose DAL (210 mg/kg per day) (DAL-L) and high-dose DAL (420 mgkg/per day) (DAL-H), compared with untreated controls (model) and sham-operated rats (sham)

**Parameter**	**Sham**	**Model**	**Captopril**	**DAL-L**	**DAL-H**
BW (g)	572.21 ± 84.23	586 ± 110.32	456.21 ± 44.46^#^	546.21 ± 74.12^△^	552.43 ± 47.87^△^
HW (g)	1.46 ± 0.17	2.0 ± 0.27*	1.50 ± 0.17^#^	1.47 ± 0.22^#^	1.62 ± 0.19^*,#,△^
LHW (mg)	268.2 ± 0.03	396.2 ± 0.09*	239.3 ± 0.02*,^#^	300.3 ± 0.04*,^#^	310.6 ± 0.03^*,#,△^
HW/BW	2.55 ± 0.45	3.56 ± 0.72*	3.05 ± 0.44*,^#^	2.9 ± 0.55*,^#^	2.41 ± 0.38^*,#,△^
LVAWTh	0.28 ± 0.03^#^	0.42 ± 0.06*	0.25 ± 0.03^#^	0.24 ± 0.04*,^#^	0.23 ± 0.03^*,#,△^

*Abbreviations*: *BW* body weight, *HW* heart weight, *HW/BW* heart weight to body weight ratio, *LHW* left heart weight, *LVAWTh* left ventricular anterior wall thickness.

Values are mean ± s.e.m.

*P < 0.05 vs. sham; ^#^P < 0.05 vs. model; ^△^P < 0.05 vs. captopril.

### Measurement of blood pressure

Systolic blood pressure (SBP) and diastolic blood pressure (DBP) were measured by the tail-cuff method (BP-6 noninvasive Electro-Sphygmomanometer, Chengdu Taimeng Science and Technology, Chengdu, PR China) in awaken rats. Each value was the average of three consecutive readings. The arterial systolic and diastolic blood pressure was measured at the weekend and continuous measurement was carried out three times as the average blood pressure for weeks. Weight was measured once a week.

### Measurement of left ventricular hypertrophy and cardiac mass index

The heart and 3 cm of left ventricular hypertrophy were removed. The cardiac mass index was the ratio of the rat heart weight to the body weight. The left heart weight and left ventricular anterior wall thickness were also measured.

### Assessment of the renal function

At the last week of the experiment, the animals were placed in individual metabolic cages and acclimatized for two consecutive days before a 24 h urine collection. Creatinine and urea were measured with a commercial enzyme-linked immunosorbent assay (WAK Chemie, Bad Soden, Germany) as described by the manufacturer.

### Determination of SOD activity and lipid peroxides level (MDA)

Superoxide dismutase (SOD) activity and the malondialdehyde (MDA) level were determined according to the instructions on the kit (Jiancheng Institute of Biotechnology Nanjing, China).

### Determination of plasma angiotensin-II and endothelin (ET-1)concentration

After the last measurement of blood pressure, the 2K1C rats were fasted for 12 h and anesthetized with sodium pentobarbital (30 mg/kg BW, intraperitoneally). Blood samples (8 ml) were taken from the abdominal aorta and handled as follows: 4 ml were collected on aprotinin (40 ml) and 10% EDTA disodium (30 ml), then centrifuged at 4°C, at 3000 r.p.m. for 10 min and the plasma obtained was aliquoted and frozen for biochemical analysis. Plasma Ang-II and endothelin (ET-1) concentrations were determined by using commercially available radioimmunoassay kit (Clinical Assay, Beijing, PR China).

### Statistical analysis

The values are expressed as mean value ± standard deviation (SD). The data were evaluated by using the SPSS (version 12.0) and one-way ANOVA, followed by Bonferroni *t*-test. Statistical significance was considered when value of P < 0.05.

## Results

### DOT normalized the blood pressure of 2K1C hypertensive rats

**Figure 1:**
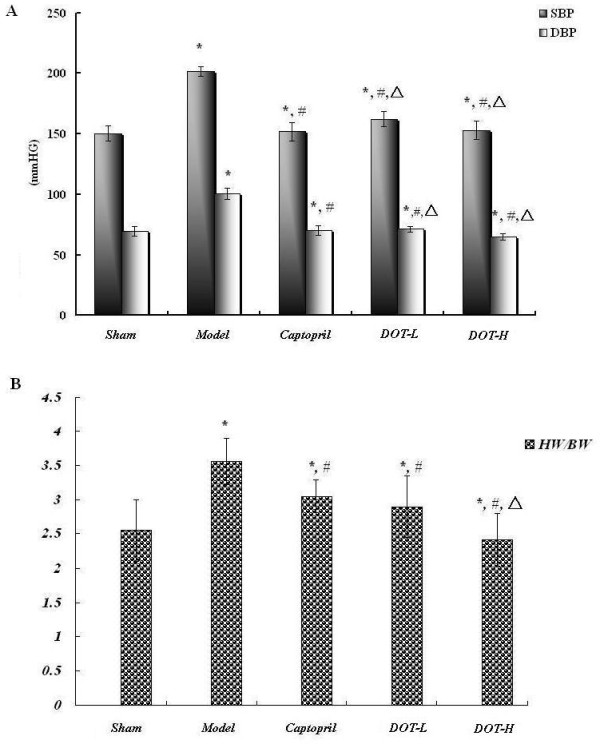
**Effect of the aqueous extract of DOT on systolic (SBP), diastolic (DBP) blood pressure (A) and heart weight to body weight ratio (B) in 2K1C hypertensive rats.** Values are mean ± SD, n = 10, *P < 0.05 vs. sham; #P < 0.05 vs. model; △P < 0.05 vs. captopril.

Untreated (model) renovascular hypertensive rats had markedly higher SBP and DBP than the sham-operated rats (Figure 1A). This hypertension was stable over 6 weeks of the experiment. In the captopril- and DOT-treated groups, the blood pressure, which was initially the same as that of the hypertensive controls, decreased progressively over the course of the 6 weeks treatment (P < 0.05) and there was no difference between the two DOT dose groups (DOT-L and DOT-H). Blood pressure was reduced by captopril to a same extent as DOT-L and DOT-H, although it was still significantly greater than in sham-operated rats.

### DOT significantly improved left ventricular hypertrophy and cardiac mass index in 2K1C hypertensive rats

In the DOT and captopril-treated groups, heart weight, left heart weight, left ventricular anterior wall thickness, heart weight/body weight ratio were lower than those of the untreated model group (p < 0.05). The values in the DOT groups (DOT-H and DOT-L) were not dose dependent. The heart weight to body weight ratio in the different dose group for DOT (DOT-H and DOT-L) was not different from that of the sham-operated rats (Figure 1B).

### DOT improved renal dysfunction effect of 2K1C hypertensive rats

**Figure 2:**
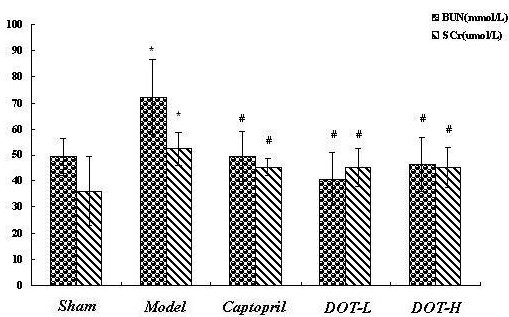
**Effect of the aqueous extract of DOT on renal function parameters in 2K1C hypertensive rats.** Values are mean ± SD, n = 10,*P < 0.05 vs. sham; #P < 0.05 vs. model.

The levels of blood urea nitrogen (BUN) and serum creatinine (Scr) were significant higher in the untreated model group than in the other group (P < 0.05). However, there was no difference between the captopril-and DOT-treated groups. Both DOT-L and DOT-H were able to decrease the serum BUN and Scr levels (P < 0.05), respectively (Figure 2).

### DOT significantly regulated the antioxidant status of 2K1C hypertensive rats

**Figure 3:**
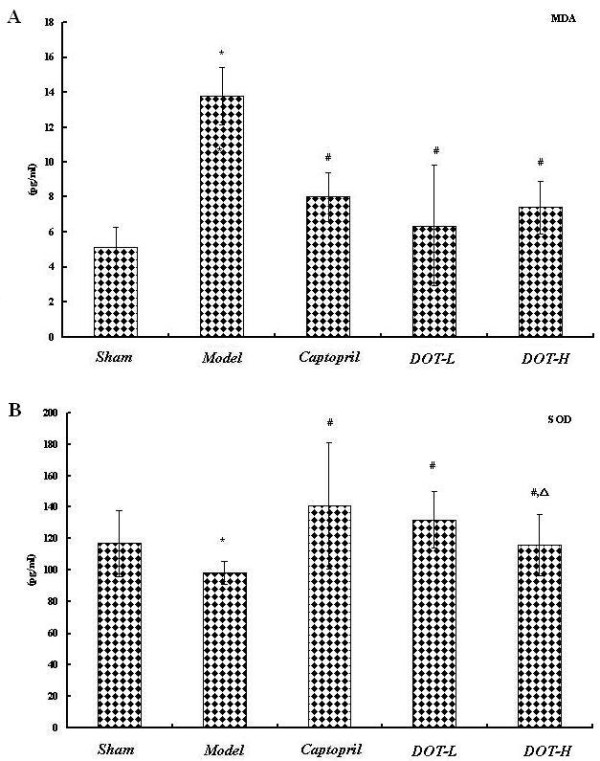
**Effect of aqueous extract of DOT on (A) plasma superoxide dismutase (SOD) and (B) plasma malondialchehyche (MDA) in 2K1C hypertensive rats.** Values are mean ± SD. n = 10, * p < 0.05 vs. sham; # P < 0.05 vs. model; △P < 0.05 vs. captopril.

The plasma SOD activity in the untreated control group was significantly lower than that in the sham-operated group. The SOD activity of the DOT and captopril-treated groups was significantly higher than that in the untreated control group (P < 0.05). The DOT and captopril-treated groups showed significantly lower MDA level (P < 0.05) than the untreated model group (Figure 3A,B).

### DOT inhibited serum Ang-II and ACE activity of 2K1C hypertensive rats

**Figure 4:**
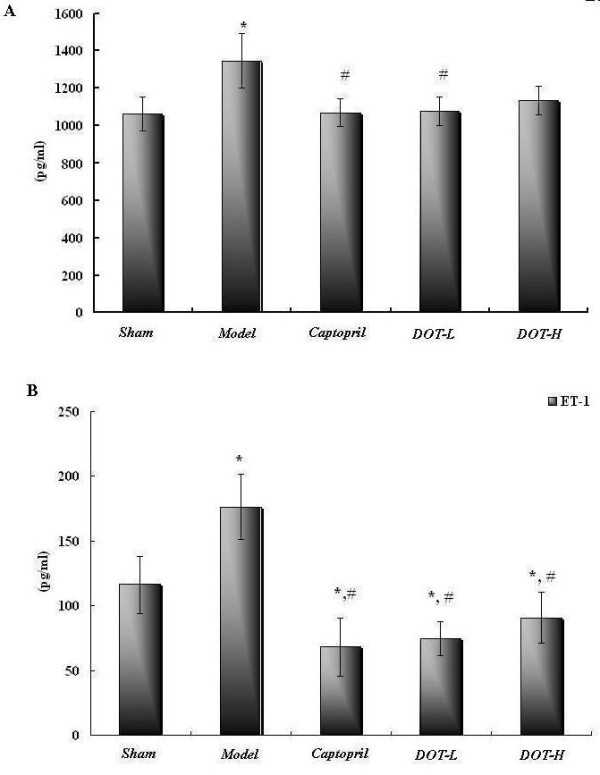
**Effect of the aqueous extract of DOT on (A) plasma angiotensin-II (Ang-II) and (B) plasma endothelin-1(ET-1) concentrations (pgml-1) in 2K1C hypertensive rats.** Values are mean ± SD. n = 10,*P < 0.05 vs. sham; #P < 0.05 vs. model; △P < 0.05 vs. captopril.

The changes in ET-1 and Ang-II in each group are shown in Figure 4. Both ET-1 and Ang-II were higher in the untreated control group than in the sham-operated group. Both ET-1 and Ang-II in DOT-H and DOT-L were lower than those in the untreated control group (P < 0.05) and even lower than those in the sham-operated group (Figure 4A,B). Ang-II level was higher in the untreated control group than in sham-operated group, but returned to about the same level as in the sham-operated group after treatment with captopril and DOT-H.

## Discussion

The 2K1C renovascular hypertension is a classic animal model of renin-dependent hypertension, which is considered to be similar to human renal hypertension. The 2K1C hypertension is an Ang-II dependent model of hypertension where increased plasma and intrarenal Ang-II concentrations [32,33], enhanced production and systemic delivery of Ang-II by the clipped kidney, form the basic endocrine disturbance [34]. The endothelin (ET) system and RAS are two of the most potent vasopressor mechanisms identified to date and in conditions where both systems are activated, their interrelationships have been proposed to contribute to the development of hypertension [35]. Previous reports showed that blood pressure regulation is dependent on the relationship between the ET system and RAS [36]. The active component of the RAS is Ang-II, which is a potent vasoconstrictor and the mechanism of RAS-induced hypertension has generally been attributed to the direct vasoconstrictor effects of Ang-II and the mineralocorticoid effects of aldosterone. Ang-II can also elevate blood pressure by augmenting noradrenaline release from sympathetic nerve endings in the vasculature [37] by increasing secretion of potent vasoconstrictor ET-1. Besides, Ang-II is also the most potent dipsogen, which could induce an increase in blood volume. On the other hand, ET-1, a major peptide primarily produced by endothelial cells, is preferentially released toward vascular smooth muscle cells to produce sustained increases in vascular tone. It is also a key factor contributing to the extracellular volume and blood pressure homeostasis. Alterations of the ET-1 system have been documented in renal diseases in which cardiovascular disorders including hypertension and endothelial dysfunction coexist [38]. Thus, in these pathological conditions, ET-1 has been implicated in the development of hypertension, cardiovascular hypertrophy and consequently, the progressive decline in the renal function. Previous findings suggested that the involvement of ET-1 in the pathogenesis of hypertension may be secondary to other factors despite the fact that ET-1 plays a determinant role in the vascular and renal damage. Research results demonstrated that there is a link between ET-1 and Ang-II. Ang-II is an important endogenous modulator of ET-1 production [39,40] and a powerful stimulator of ET-1 formation in VSMCs and endothelial cells [41,42], which can further increase vascular contractile response to Ang-II. Moreover, the hypertensive and renal vasoconstrictor effects together with the natriuresis and diuresis actions of Ang-II are mediated by ET-1 [43]. Chronic infusion of suppressor doses of both Ang-II and ET-1 was shown to induce significant increase in SBP. The results of the present study show that DOT could decrease hypertension in this Goldblatt 2K1C model. The effects of high (DOT-H) and low (DOT-L) doses were not significantly different from that of captopril. Furthermore, there was no dose dependence of this effect which could be already maximal for this extraction. This hypertensive effect was accompanied by a significant decrease of the plasma levels of ET-1 and Ang-II. Although the ET-1 level of DOT-L and DOT-H treated groups were found to be higher than that of captopril-treated group, the level of Ang-II of the DOT-L group was nearly the same as that of the captopril-treated group. Interestingly, while DOT caused only small effect on Ang-II, both doses of DOT (DOT-L and DOT-H) were found to reduce significantly the ET-1 concentration to lower values than that in the sham-operated group, being the ET-1 value of the low dose (DOT-L) less than half of the untreated control group. These results suggest that DOT might act at least in part by reducing the ET release in a manner that might be associated with the ET-converting enzyme inhibitors. The major finding in this study is that administration of DOT attenuated cardiac hypertrophy in the 2K1C hypertension rats, and in combination with reduced blood pressure may explain an attenuation of cardiomegaly. Since we investigated the effect of DOT on cardiac function, we also observed the reduction in the heart weight which indicated an improvement in cardiovascular function parameters. The different effect of DOT on Ang-II and ET-1 is reflected by the effect on myocardial hypertrophy which is one of the major end points in the treatment of hypertension. Even though the values of the SPB and DPB of the DOT-H treated group were very similar to those of the captopril-treated group, the heart weight to body weight ratio of the DOT-H treated group was lower than that of the captopril-treated and the sham-operated groups. The dissociation between the effect of blood pressure and myocardial hypertrophy found in DOT treated group is not surprising since this effect has already been described [22]. Although the captopril-treated rsts had lower absolute heart weight, left heart weight and anterior wall thickness than the DOT-treated rats, they also had lower body weight. Consequently, the effect of captopril on the heart weight to body weight ratio was higher than DOT. In addition, there was a tight linear correlation between heart weight and blood pressure level, indicating that the development of cardiac hypertrophy was entirely dependent on the development of hypertension [44,45]. Inhibition of ET production which resulted in inhibition of ET induced vasoconstriction and mitosis can also inhibit formation of hypertension and cardiac hypertrophy.

A recent hypothesis pointed out a possible role of oxidative stress as a key player in the pathogenesis of insulin resistance, cell dysfunction, and hypertension [46,47] and many mechanisms have been implicated in processes underlying oxidative stress-mediated hypertension. The relationship between the development of hypertension and the increased bioavailability of ROS or decreased antioxidant capacity, or both, have been demonstrated in many experimental models of hypertension [48] as well as in human hypertension [49]. These findings are based, in general, on increased levels of biomarkers of lipid peroxidation and oxidative stress [50]. Experimental and clinical studies suggest that oxidative stress precedes the manifestation of elevated blood pressure and this may be one of the initiating factors promoting progression from the pre-hypertensive stage to the hypertensive stage [51]. Research data suggest that oxidative damage and pro-inflammatory processes may precede full-blown hypertension [52]. The “ROS family” comprises many molecules that have divergent effects on cellular function, such as regulation of cell growth and differentiation, modulation of extracellular matrix production and breakdown, inactivation of NO, and stimulation of many kinases and pro-inflammatory genes. Furthermore, ROS can promote inflammation, alteration of vasomotion and activation of matrix metalloproteinases, thus causing platelet aggregation and stimulation of vascular smooth muscle proliferation [53,54]. Decreased antioxidant activity (SOD and catalase) and reduced levels of ROS scavengers may also contribute to oxidative stress. A large number of clinical studies and animal experiments indicated that there is an increase in oxidative stress and generation of oxygen free radicals (OFR) but significantly reduction of SOD activity in the pathophysiology of hypertension status. A large number of OFR can cause lipid peroxidation, thereby undermining the cell membrane unsaturated fatty acids by forming lipid peroxides (LPO). As malondialdehydes (MDA) are a class of terminal lipid peroxidation metabolites, determination of their content can directly reflect lipid peroxidation levels. On the other hand, SOD is a natural antioxidant enzyme which can remove superoxide anion radicals *in vivo* to maintain the production of free radicals in the body and clear the dynamic equilibrium of a metal enzyme. Therefore, protection of the endothelial function and reduction of free radical damage in the treatment and prevention of hypertensive target organ damage can have a far-reaching significance. No matter if the oxidative stress is indeed a cause or consequence of hypertension, a decrease in oxidative damage may result in a reduction in blood pressure. As antioxidants are compounds that are capable of trapping ROS, they may be able to reduce oxidative damage, and possibly blood pressure. Our results showed that the plasma SOD activity in the untreated control group was lower than that in the DOT-H, DOT-L and Captopril-treated groups. On the contrary, MDA content in the untreated control group was significantly higher (p < 0.05) than that of the DOT-H and DOT-L-treated groups. The results obtained suggest that DOT may play a key role in scavenging OFR, thus causing anti-oxidation effects and can clear free radicals from the system, which in turn effectively improved the endothelial lipid oxidative damage and high blood pressure to the same extent as captopril, thus guaranteeing its therapeutic effects.

## Conclusion

The present study demonstrates significant antihypertensive effects, which are mainly characterized by reduction in blood pressure and prevention of left ventricular hypertrophy, of DOT. We hypothesized that DOT may prevent cardiac hypertrophy by reduction of Ang-II level, inhibition of E-1 production, and induction of the *in vivo* antioxidant defense system. These findings could provide a basicly scientific explanation to not only the widespread use of herbal preparations derived from *Dioscorea opposite* Thunb. in TCM in China but also a beneficial effect of yam in food products which could be considered useful information for food processing in the development of functional food for blood pressure regulation. Nevertheless, further toxicological and pre-clinical studies should be evaluated in order to warrant the therapeutic use of this extract for combating hypertension.

## Competing interests

The authors have no actual or potential conflict of interest associated with this work.

## Authors’ contributions

NA, RA, SA, DM and AK participated in the design of the study data analyses and manuscript preparation. PH and ZO conducted the assays and analyses. All authors read and approved the final manuscript.

## Pre-publication history

The pre-publication history for this paper can be accessed here:

http://www.biomedcentral.com/1472-6882/14/36/prepub
